# Supplementary material for: Learning curve and surgical outcome of robotic assisted colorectal surgery with ERAS program
Source: Sci Rep. 2022 Nov 29;12:20566. doi: 10.1038/s41598-022-24665-w (PMC9709162; doi:10.1038/s41598-022-24665-w)
Supplement: Supplementary file 1 — Supplementary Information 1. [file 41598_2022_24665_MOESM1_ESM.zip › LSC Raw data-Ñ[▒K/057/HS_ñw╝╨░O▒Kñσ.pdf]

正式摘要(電子簽章：00000018333092)

W55 / W55 ☐英文標題(注意:無英文姓名-請補病人基本檔!) ☐【讀取】憑證

臺中榮民總醫院

出院病歷摘要

轉入號： 轉出號：

[redacted]

(8)流水編號	
(9)入院日期	108/12/13 (2019) 直腸外科 W55 - 121
(10)轉科日期	
(11)出院日期	108/12/19 (2019) 住院天數計 6 日

診斷

- 入院 :Upper rectal cancer
- 出院 :Upper rectal cancer, s/p laparoscopic Low Anterior resection on 20191214, cT1N0M0.

主訴

- Bloody stool noticed for weeks

病史

- This 59 years old male patient denied other underlying disease. He noticed that he had bloody stool for weeks. He denied fever, appetite change, weight loss, bowel habit changes. So he came to our OPD and recieved sigmoidoscopy on 12/12, and it showed COLON TUMOR WAS NOTED AT 10CM FAV, S/P BIOPSY. Rectum cancer was suspected.
  As mentioned above, he was admitted to our ward for surgery.

體檢發現

-
  - General appearance :
    - easy looking , conscious clear , without cardiopulmonary distress
    - Vital signs自動帶入date 體溫(BT) 脈搏(P) 呼吸(R) 血壓(BP) 身高(BH) 體重(BW) BMI
      20191213 36℃ 95次/分 20次/分 169/96mmHg 166.3cm 80.2kg 29.0
    - Vital signs: B.P. 169 mmHg , P.R. 95 /min , R.R. 20 /min , B.T. 36 ℃
  - HEENT :
    - Eyes: no anemic conjunctiva, no icteric sclera, normal EOM
    - ENT: no otorrhea, no rhinorrhea, no erythematous mucosa, no injected tonsil, no deformity
  - Neck: supple, freely movable, trachea midline,no deformity, no engorged jugular vein , no palpable thyroid or lymph node enlargement
  - Chest:
    - Inspection: Grossly normal, symmetric and fully expansion, no accessory muscle used, no operation scar, no spider angiomata, no gynecomastia
    - Palpation: no palpable mass or nodule, no subcutaneous emphysema, no tenderness
    - Ausculation: clear breathing sound
  - Heart:
    - Palpation: no thrill, no heave
    - Percussion: normal heart size
    - Ausculation: regular heart beat, normal S1, S2, no gallop, no murmur
  - Extremities: No deformity, full ranged of motion without limitation, no clubbing finger or nail cyanosis, no pitting edema, no ulcer or pigmentation, no obvious skin lesion, no varicose vein, no bone, joint or muscle tenderness

  CRS CONDITION

  - Karnofsky performance status: (100) Normal, no complaints, no evidence of disease
  - PE of Abdomen:
  - CEA: 1.49 ng/ml
  - Imaging Studies:
  - Colonoscope report: COLON TUMOR WAS NOTED AT 10CM FAV, S/P BIOPSY.
  - Figures:
    1. colon & rectum

手術日期及方法

- 手術日期及方法20191214 1. Laparoscopic low anterior resection with end-to-side anastomosis, endo-GIA 60/4.1mm*2, CEEA 33mm, re-inforcement with silk: nil, bilateral doughnuts: intact, air leak test: done, no air leak.
  2. High ligation (+): IMA above left colic artery.
  3. Cut margin: proximal 1cm, distal 20cm.
  4. Reperitonealization: nil.
  5. Take down of splenic flexure: nil.
  6. One 12Fr. Penrose drain at pelvis.
- 手術結果及發現 :
  - 1. A 1x1cm ulcerative Borrmann's type I tumor was found at upper rectum, s/p clip mapping, which limited in submucosa and mucosa layer. on enlarged indurated lymph nodes at mesocolon. Clinical stage: T1N0M0. 2. No synchronous tumor or polyp was found. 3. There was no visible liver metastasis, and no visible peritoneal seeding.

住院治療經過

- After admission, he received Laparoscopic low anterior resection with end-to-side anastomosis on 2019/12/14.
  We keep follow up his vital signs and clinical symptoms.
  Analgesic pain control were given. He try water on 12/16 without complications.
  He can eat semiliquid diet on 12/17. Due to stable condition, we remove his penrose drain and let himdiscahrge on 12/19.

合併症

一般檢查

- 累積報告：

DATE	TIME	ALB	TP	BIL.T	BIL.D	ALKP	AST	ALT	LDH	CHOL	TG	HDL-C	LDL	UA	NH3	AMY
1081213	1434	4.0		0.8	0.2	85	20	19	154	195		37	143			
1081216	0912						17	12								

DATE	TIME	WBC	RBC	HGB	HCT	MCV	PLT	BLST	NEUT	BAND	SEG	LYM	MON	EOS	BAS
1081213	1437	6610	4.60	13.2	40.0	87.0	221		57.7			29.0	7.1	5.3	0.9
1081215	0027			12.2											
1081216	0928	7150	4.09	11.9	36.1	88.3	235		75.9			15.7	6.3	1.7	0.4

DATE	TIME	NA	K	CL	CA	BUN	CREAT	Mg	P	CRP	ESR	PCT	LIPASE	LACTATE
1081213	1434	142	3.5		8.9	18	1.19			1.025				
1081216	0912	138	3.7			14	1.06							

DATE	TIME	GLUCOSE	PAB	TRF	ZN	CU	KETONE	CK	CKMB	PT-P	PT-C	APTT-P	APTT-C	TRO-T
1081213	1434	101												
1081213	1437									10.4	10.1	28.4		

- 放射線報告 :檢驗項目 **:CT ABDOMEN-SURVEY** 報告日期 **:1081219**

主　訴：
Result:
Finding:
Imaging Report Form for Colorectal Carcinoma (AJCC 8th)
1. Imaging Date: 20191213
Imaging Modality, Imaging by
CT scan
Imaging Protocol:
Post-contrast computed tomography scan of whole abdomen was performed with oral contrast ingestion
2. Tumor location / Size
Location:
Rectum
Size:
Measurable: 3 cm (largest diameter)
3. Tumor invasion
Tumor within colonic or rectal wall
4. Regional nodal metastasis
No
5. Distant metastasis (In this study)
No or Equivocal
6. Other findings:
> Focal irregular wallthickening over rectum(ser401, img83) is noted. Rectal cancer is considered. Suggest clinicial correlation.
> Low attenuation lesion about 1.2cm in size over lateral segment of liver, in favor of cyst. Suggest close follow up or further evaluatoin.
> Another small low attenuation lesion over S8 of liver. In favor of cyst. Suggest F/U.
> Prominence of left adrenal gland
> No definite focal lesion in spleen, pancreas, gallbladder, and kidneys.
> No significant lymphadenopathy in the abdomen or retroperitoneum identified.
> Degenerative change of lumbar spine with osteophyte formation
Impression:
R/O rectal cancer, cT1-2N0M0

特殊檢查

病理報告

- pending

其它

出院時情況

- 3.改門診治療

出院指示

-

藥名	劑量	劑量單位	用法	頻次	數量	數量單位	現況	天數	單位
Acetylcysteine tab	1	TAB	PO	BID	10	TAB	TH	5	天

出院計劃

- 門診日期
  - date on 2020/01/07
- 衛教 :Post op wound care
- Arrange OPD follow up

主治醫師 2062F 蔣鋒帆 / 住院醫師 2339C 蔡濬樑

正式摘要(電子簽章：**000000183333092)**

W55 / W55

doctorRank:B
